# Quantitative prediction model for affinity of drug–target interactions based on molecular vibrations and overall system of ligand-receptor

**DOI:** 10.1186/s12859-021-04389-w

**Published:** 2021-10-14

**Authors:** Xian-rui Wang, Ting-ting Cao, Cong Min Jia, Xue-mei Tian, Yun Wang

**Affiliations:** Key Laboratory of TCM-Information Engineer of State Administration of TCM, School of Chinese Pharmacy, Beijing University of Chinese Medicine, Beijing, 100102 China

**Keywords:** Molecular vibrations, Random forest, Drug–target affinity, Chemical composition, Drug–target interactions

## Abstract

**Background:**

The study of drug–target interactions (DTIs) affinity plays an important role in safety assessment and pharmacology. Currently, quantitative structure–activity relationship (QSAR) and molecular docking (MD) are most common methods in research of DTIs affinity. However, they often built for a specific target or several targets, and most QSAR and MD methods were based either on structure of drug molecules or on structure of receptors with low accuracy and small scope of application. How to construct quantitative prediction models with high accuracy and wide applicability remains a challenge. To this end, this paper screened molecular descriptors based on molecular vibrations and took molecule-target as a whole system to construct prediction models with high accuracy-wide applicability based on dissociation constant (Kd) and concentration for 50% of maximal effect (EC50), and to provide reference for quantifying affinity of DTIs.

**Results:**

After comprehensive comparison, the results showed that RF models are optimal models to analyze and predict DTIs affinity with coefficients of determination (R^2^) are all greater than 0.94. Compared to the quantitative models reported in literatures, the RF models developed in this paper have higher accuracy and wide applicability. In addition, E-state molecular descriptors associated with molecular vibrations and normalized Moreau-Broto autocorrelation (G3), Moran autocorrelation (G4), transition-distribution (G7) protein descriptors are of higher importance in the quantification of DTIs.

**Conclusion:**

Through screening molecular descriptors based on molecular vibrations and taking molecule-target as whole system, we obtained optimal models based on RF with more accurate-widely applicable, which indicated that selection of molecular descriptors associated with molecular vibrations and the use of molecular-target as whole system are reliable methods for improving performance of models. It can provide reference for quantifying affinity of DTIs.

**Supplementary Information:**

The online version contains supplementary material available at 10.1186/s12859-021-04389-w.

## Background

The rapid development of systems biology has proposed a new view that a single drug molecule acts on multiple targets or that multiple drug molecules act on a common target [1, 2]. That is to say, there are multiple interactions between targets and drug molecules-DTIs. DTIs plays an important role in pharmacology, biology and mechanism [3–6]. For example, on the basic of DTIs research, the off-target toxicity of appetite suppressant Fen-Phen that can cause death is due to the activation of 5-HT2B receptor by one of its metabolites-Norfenfluramine, leading to proliferative valvular heart disease [7]. In study of repositioning salicylanilide anthelmintic drugs to treat adenovirus infections, the results showed that Niclosanide and Rafoxanide target transport of HAdV particle from endosome to nuclear envelope, whilst oxyclozanide specifically targets adenovirus immediately early gene E1A transcription [8]. Therefore, the research of DTIs will help to understand mechanisms or toxic side effects of drugs and repositioning of drugs [9–12].

Currently, the research on DTIs focused on two directions, one is traditional experimental analysis and the other is DTIs predictive analysis based on existing databases [13]. Traditional experimental analysis of DTIs is expensive and inefficient, and faces many challenges such as financial, technical and time aspects. It is almost impossible for researchers to carry out experiments to identify mechanisms or toxic side effects for all drug compounds. In comparison, the prediction of DTIs that is efficient and low cost can make up for shortcomings of traditional trials [14]. In prediction of DTIs, prediction of drug–target affinity is becoming increasingly important. This is because prediction of affinity not only predicts weather there is an interaction between molecules and targets, but also obtains strength of interaction, which is useful for drug discovery, effect and toxic evaluation, etc. Computational approaches for DTIs affinity in most of current research mainly include two categories: ligand-based and receptor-based methods [15, 16]. In above methods, quantitative structure–activity relationship (QSAR) and molecular docking (MD) are most common methods. Such as Simeon S, et al., constructed QSAR models of Janus kinase 2 inhibitors based on machine learning algorithms to predict inhibitory potency [17]. Luo M, et al., used random forests (RF), support vector machine (SVM), and K Nearest Neighbors (KNN) to construct QSAR models of 5‑HT1A Receptor, in which Ki value characterized affinity of receptor-ligand [18]. Van Den Driessche G and Fourches D used 3D molecular docking to reveal common HLA-B*57:01 variants that trigger adverse drug reactions [19]. In addition, there is also a similarity search-based approach, which utilizes chemical structure similarity to predict DTIs and DTIs affinity [20, 21].

However, quantitative structure–activity relationship (QSAR) and molecular docking (MD) have some limitations. QSAR or MD is often built for a specific target or several targets, making it difficult to achieve quantitative prediction for multiple targets at the same time, which leads to a small range of applications. Moreover, molecular docking and its evaluation methods are limited to 3D structure of target proteins [22–24]. Molecular docking is inaccurate when those proteins whose 3D structure is unknown, especially for membrane proteins whose 3D structure is difficult to crystallize [25, 26]. These limitations are severe because most useful drug targets are membrane proteins, such as ion channels and G protein-coupled receptors (GPCRs) [27, 28]. This leads to low accuracy and low applicability of most DTIs prediction models, not to mention prediction of affinity for DTIs. The more serious fact is that most QSAR and MD were based either only on structure of ligands or on structure of receptors. By considering only structure of receptor or ligand, similarity-based analysis inevitably leads to inaccurate results that are inconsistent with experimental results. This fragmented approach ignores holistic nature of receptor-ligand interactions, which leads to low prediction accuracy and excessive bias. In addition, in constructing quantitative prediction models, researchers mostly used molecular descriptors to solve problem of quantifying abstract molecules, and solved mapping problem of best-described function by optimizing algorithm and parameters. However, researchers ignore problem of feature characterization. This can also lead to low accuracy and excessive bias for prediction of DTIs affinity [29, 30].

In this paper, with above limitations in mind, we took molecule-target as a whole system from systems biology perspective to construct prediction models for DTIs affinity with high accuracy and wide applicability, in which simultaneously considering both receptors and ligands. Molecular descriptors associated with molecular vibrations were combined with protein sequence descriptors to construct whole system of molecule-target, in which Kd and EC50 were used as quantitative indicators. On the premise of feature selection, combining machine-learning algorithms to predict DTIs affinity efficiently and accurately. These models consisted of internal cross-validation and external tests, which provided a predicted performance with high accuracy and wide applicability. In addition, optimal models were selected for application evaluation and comprehensive comparison. The new quantitative models will provide reference for prediction of DTIs affinity.

## Results and discussions

**Fig. 1:**
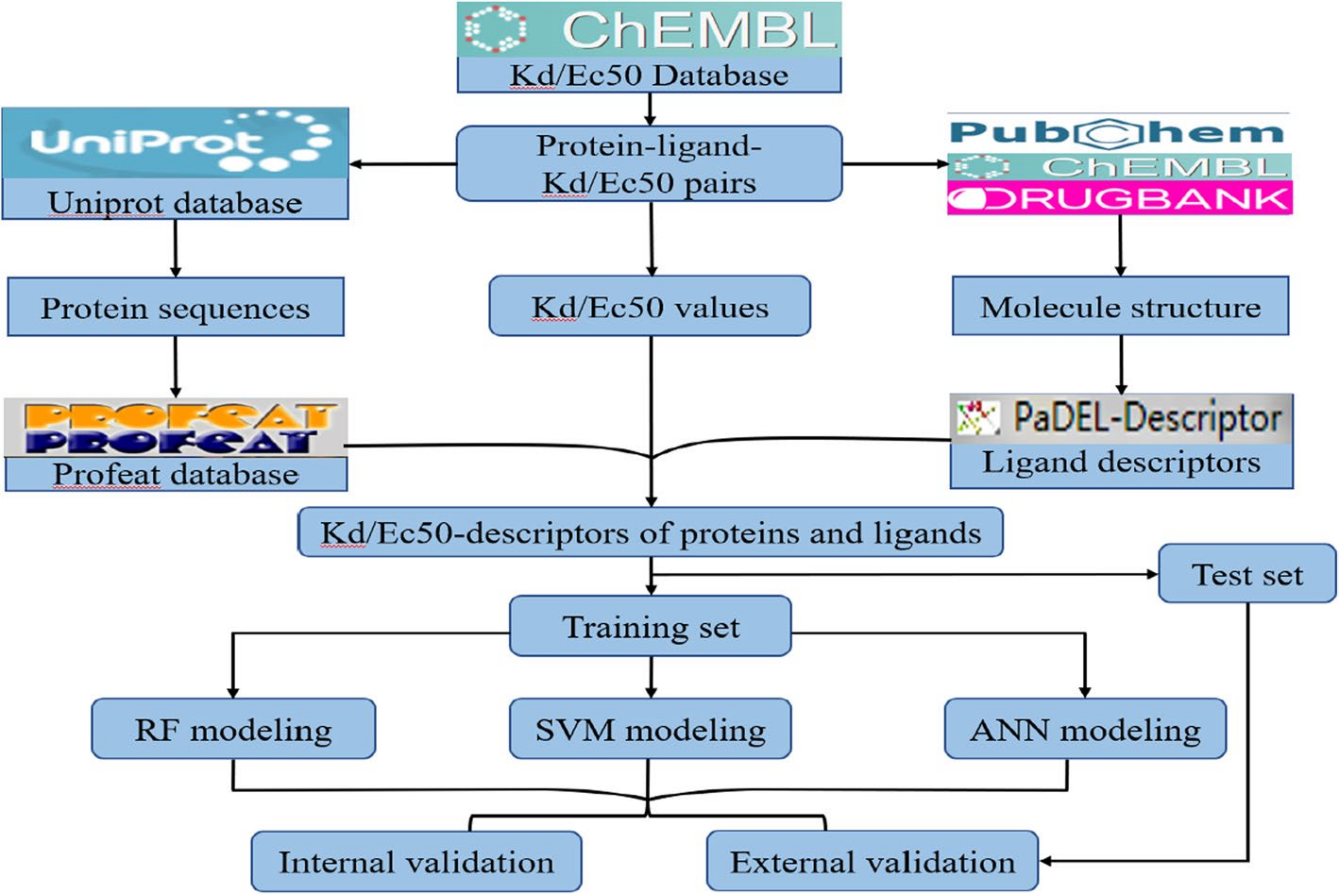
The specific research methodology

As shown in Fig. 1, this paper was completed under that research methodology. In the following section, we presented and discussed the data collection, results of descriptor calculation, optimal prediction models, the importance of descriptors and so on.

### Data collection

Based on open source database: PubChem (https://pubchem.ncbi.nlm.nih.gov/), Drugbank (https://go.drugbank.com/), ChEMBL (https://www.ebi.ac.uk/chembl/) and Uniprot database (https://www.uniprot.org/), we performed data collection of drug molecules, target protein sequences, and Kd and EC50 values characterizing drug molecule-target affinity. Taking drug molecule and target as a whole system, we obtained the EC50 dataset-quantifying DTIs affinity by EC50 and the Kd dataset-quantifying drug molecule-target affinity by Kd, respectively. The EC50 dataset contains 8147 ligands and 544 targets, and 11,076 ligand-target-EC50 pairs. At the same time, The Kd dataset contains 1870 ligands and 778 targets, and 10,923 ligand-target-Kd pairs. The two datasets without redundancy were used as benchmark datasets.

In process of data collection, we kept to the following two criteria: (1) maintain entries as many as possible; (2) exclude redundant data as many as possible. Therefore, some drug molecules and targets were removed due to Kd, EC50 has no definite value, or their activity values are inconsistent. These redundant data may strongly affect the accuracy of prediction models for DTIs affinity. It is worth noting that EC50 refers to the concentration of a drug, antibody or toxicant that achieves 50% of the maximum biological effect after a specified exposure time. It was commonly used as a measure of a drug's potency [31]. Kd is often used to describe degree of binding of a compound to a particular target [32]. The smaller dissociation constant, the higher affinity between compounds and proteins. Considering the practical significances of Kd and EC50, we finally chose both as quantitative indexes of DTIs affinity.

### Results of descriptor calculation

#### Calculation of drug molecule descriptors

After calculation by online platform-PaDEL, we obtained the molecular descriptors. The descriptors calculated in this article were shown in Table 1. There were 1874 descriptors for drug molecules and drug molecular descriptors can be divided into 16 categories, among which E-state descriptors, Autocorrelation descriptors and Topological type descriptors account for a relatively large number. Even though many descriptors in Table 1 are of the same type, each descriptor has its own specific meaning. However not all molecular descriptors are suitable for the construction of predictive models for DTIs affinity.

**Table 1:** Type and number of drug molecule descriptors

Serial number	Descriptor type	Number of descriptors
1	Constitutional descriptors	120
2	Autocorrelation descriptors	346
3	Basak descriptors	42
4	BCUT descriptors	6
5	Burden descriptors	96
6	Connectivity descriptors	56
7	E-state descriptors	489
8	Kappa descriptors	3
9	Molecular property descriptors	15
10	Quantum chemical descriptors	5
11	Topological descriptors	265
12	CPSA descriptors	29
13	RDF descriptors	210
14	Geometrical descriptors	21
15	WHIM descriptors	91
16	3D Autocorrelation descriptors	80

Therefore, how to measure importance of descriptors and filter out meaningful ones is the key to improve accuracy of prediction models. Some researchers used kernel functions, thresholds, and other methods to filter descriptors to improve accuracy of models [33, 34]. It is worth considering that these methods don’t take into account properties of drug molecules and that may not be applicable in quantitative prediction of DTIs.

After comprehensive consideration, in this paper, based on properties of drug molecules, we screened characteristic descriptors of drug molecules from the perspective of molecular vibrations. This is because molecular vibrations are caused by vibrations of chemical bonds within molecules and they are macroscopic representation of drug molecules properties [35, 36]. Moreover, molecular vibrations are affected by various factors such as conjugation effect, induction effect, spatial effect, hydrogen bonding, vibrational coupling effect, etc. Therefore, molecular vibrations can reflect drug molecular structure and physicochemical properties of drugs to a certain extent [37]. It should be remembered that seven physicochemical properties are particularly relevant to molecular vibrations, including electronegativity, π-atomic charge, total charge, and bond polarity [38]. Therefore, we choose molecular descriptors related to molecular vibrations based on above physicochemical properties. For instance, Mpe-Constitution Descriptor-mean Atomic Pauling Electronegativity (scaled on carbon atom) was selected as feature descriptor to construct prediction models for DTIs affinity due to its relation to atomic electronegativity. Finally, 813 descriptors associated with molecular vibrations were selected from 1874 descriptors in Table 1 to represent the feature characteristics of drug molecules. In addition, 813 molecular descriptors associated with molecular vibrations and their specific meanings were given in Additional file 3: Table S1.

#### Calculation of target protein descriptors

As was known to all, 3D structures of many proteins are unknown, especially for membranous proteins [27, 28]. Thus, the analysis based on protein sequences rather than 3D structures of proteins can ensure a wide range of applicability of models and accuracy [39]. The target protein descriptors were shown in Table 2.

**Table 2:** Type and number of target protein descriptors

Serial number	Descriptor type	Number of descriptors
G1	Amino acid composition	20
G2	Dipeptide composition	400
G3	Normalized Moreau-Broto autocorrelation	240
G4	Moran autocorrelation	240
G5	Geary autocorrelation	240
G6	Composition	21
G7	Transition, distribution	126
G8	Sequence-order-coupling number	60
G9	Quasi-sequence-order descriptors	100

As shown in Table 2, there are 1437 descriptors for each protein and the descriptors can be divided into 9 categories, among which Dipeptide composition, Moran autocorrelation as well as Normalized Moreau-Broto autocorrelation account for a relatively large number.

813 drug molecule descriptors were integrated with 1437 protein descriptors and Kd, EC50 datasets to obtain the integrated Kd, EC50 datasets.

### Results of feature screening

**Fig. 2:**
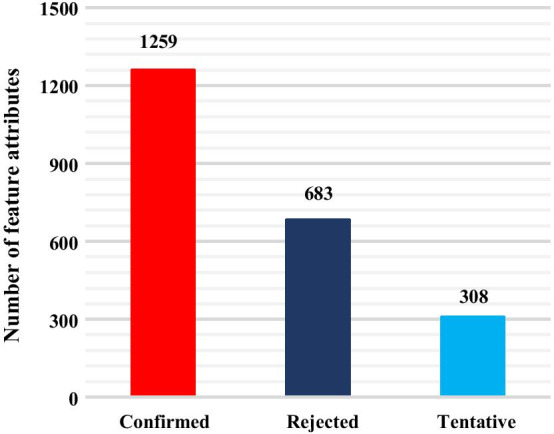
Feature filtering results for EC50 dataset

**Fig. 3:**
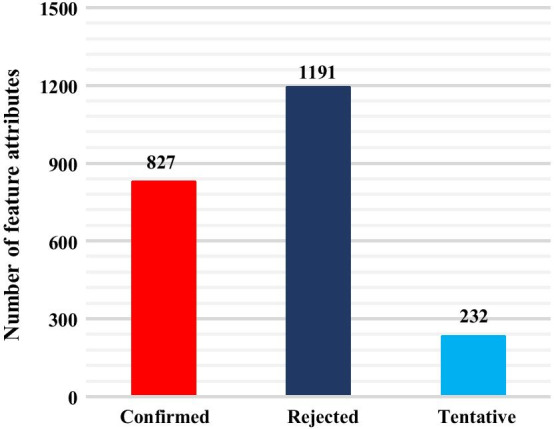
Feature filtering results for Kd dataset

The Boruta algorithm was used for feature filtering [58]. As shown in Fig. 2, for integrated EC50 dataset, 1259 descriptors were marked as “Confirmed” and 683 descriptors were marked as “Rejected”, with 308 descriptors being marked as “Tentative”. That is, after feature selection, each DTI in the integrated EC50 dataset was characterized by 1259 feature attributes. Similarly, as shown in Fig. 3, for the integrated Kd dataset, 827 descriptors were marked as “Confirmed” and 1191 descriptors were marked as “Rejected” with 232 descriptors being marked as “Tentative”. Each DTI in integrated Kd dataset was characterized by 827 feature attributes. The feature subsets of EC50 and Kd were obtained by feature screening for construction of quantitative prediction models for DTIs affinity.

The purpose of feature selection in machine learning is to filter out features set that minimize the cost function of currently selected model. However, Boruta algorithm can use a random forest approach to select the set of all features that are relevant to the dependent variable, rather than selecting the set of features that minimizes penalty factor only for a specific model such as SVM. It can also disrupt the order of features and calculate the importance of features. Boruta algorithm can help us to understand the factors influencing dependent variable more fully and make feature selection better and more efficient. Therefore, when it’s not known upfront which algorithm is optimal, we chose Boruta algorithm for feature filtering.

### Results of quantitative prediction model for DTIs affinity

#### Parameter optimization

In the RF model, there are important parameters need to be considered, such as ntree and max depth. After comparison and optimization of several parameters, we finalized RF algorithm parameters: ntree = 500, max depth = no limitation, min samples split = 2, min samples leaf = 1, max leaf nodes = none. For SVM model, we used “Tune” function to determine the optimal parameters of SVM algorithm, with the following algorithm parameters: cost = 1000, gamma = 0.0001 [40]. In same optimization way, ANN algorithm parameters were determined: size = 2, decay = 0.1, linout = T (non-linear function), maxit = 1000, max nwts = 10,000.

#### Optimal prediction model for DTIs affinity

Before attempting to construct prediction models for DTIs affinity, EC50 feature subsets were preprocessed to facilitate calculation. Then combined with SVM, RF and ANN to construct quantitative prediction models respectively. The results of tenfold cross validation for EC50 feature subset were shown in Table 3.

**Table 3:** Tenfold cross validation of three kinds of algorithms for EC50 feature subset

Model (EC50)	R^2^		MSE		RMSE		MAE		SSE	
	Train	Test	Train	Test	Train	Test	Train	Test	Train	Test
SVM	0.9317	0.5759	0.1270	0.8356	0.3564	0.9146	0.1960	0.5801	1249	8216
RF	0.9611	0.9641	0.0891	0.0817	0.2985	0.2858	0.1976	0.1989	876	803.3
ANN	0.7350	0.5211	0.4867	0.9590	0.6976	0.9793	0.5023	0.6792	4785	9429

**Fig. 4:**
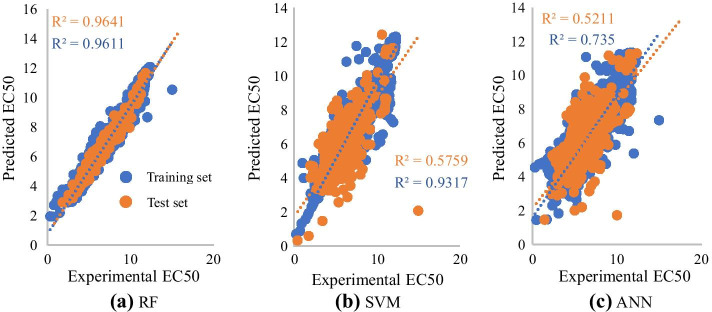
Scatter plot of experimental and predicted EC50 values for three prediction models (**a**: EC50 scatterplot based on random forest model, **b**: EC50 scatterplot based on support vector machine model, **c**: EC50 scatterplot based on K Nearest Neighbors model.)

As shown in Table 3 and Fig. 4, In RF model, R^2^ of training and test sets are 0.9611, 0.9641 respectively indicated a good fit of RF model to data. MSE of training and test sets were both less than 0.09 and were in same order of magnitude, which indicated that there is no overfitting problem existing, and demonstrated that RF model showed satisfactory predictive performance (Fig. 4a). As for SVM model, R^2^ of training and test sets are 0.9317, 0.5759 respectively. SVM model exhibited some differences between training and test sets, but order of magnitude is the same and no greatly obvious overfitting can be observed from SVM model (Fig. 4b). However, predictive performance of the SVM model was worse than that of the RF model. For training and test sets in ANN model, no obvious overfitting can be observed (Fig. 4c), but the performance of ANN model in training and test set were lower than both RF model and SVM model. By comparing predictive performance of three models based on evaluation indicators, it can be observed that the performance of RF model is best selection for EC_50_ data.

**Fig. 5:**
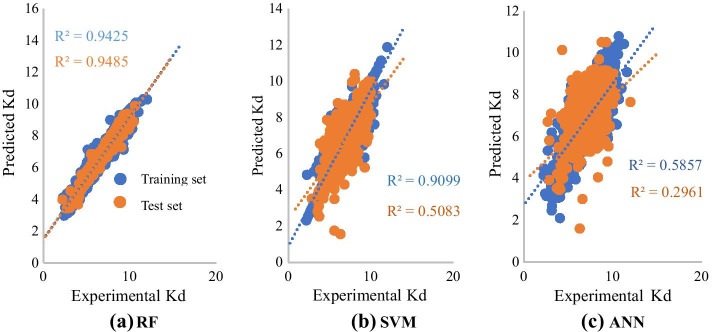
Scatter plot of experimental and predicted Kd values for three prediction models (**a**: Kd scatterplot based on random forest model, **b**: Kd scatterplot based on support vector machine model, **c**: Kd scatterplot based on K Nearest Neighbors model.)

The same analysis was appropriate for Kd dataset, on the basic of data in Table 4 and scatter plot in Fig. 5, we completed selection of optimal model: RF model showed satisfactory predictive performance with R^2^ of test set being 0.9485 (Fig. 5a). The SVM model suffered from overfitting and its predictive performance was worse than that of RF model (Fig. 5b). ANN model was the least effective model (Fig. 5c). The results indicated that RF model is the optimal quantitative prediction model for KD dataset.

**Table 4:** Tenfold cross validation of three kinds of algorithms for Kd feature subset

Model (Kd)	R^2^		MSE		RMSE		MAE		SSE	
	Train	Test	Train	Test	Train	Test	Train	Test	Train	Test
SVM	0.9099	0.5083	0.1254	0.7290	0.3541	0.8538	0.2116	0.6406	1230	808.4
RF	0.9425	0.9485	0.1208	0.1191	0.3476	0.3451	0.2640	0.2594	1204	132.1
ANN	0.5857	0.2961	0.5612	1.0190	0.7491	1.0095	0.5792	0.7390	5593	1130

In summary, whether based on EC50 dataset or Kd dataset, the performance of RF models are the best. Therefore, in this paper, random forest (RF) models are more suitable for quantitative prediction of biological activities for DTIs affinity.

### Evaluation of application for optimal models

By comparing analysis in 2.4, we obtained RF optimal models. To demonstrate the reliability and applicability of RF models further, we used RF models for analysis of DTIs in Binding DB database, in which Kd and EC50 quantified affinity of DTIs.

Using same data collection methods and eliminating duplicate data, we collected 1045 ligand-receptor-EC50 pairs and 89 ligand-receptor-Kd pairs from Binding DB database for quantitative analysis of DTIs affinity. Quantitative analysis of new dataset was carried out using RF models based on Kd and EC50. Calculating absolute value of the difference between true value and predicted value (referred as '|d|' from now) and dividing |d| into 5 parts in which each part was divided on a scale of 0.5.

**Fig. 6:**
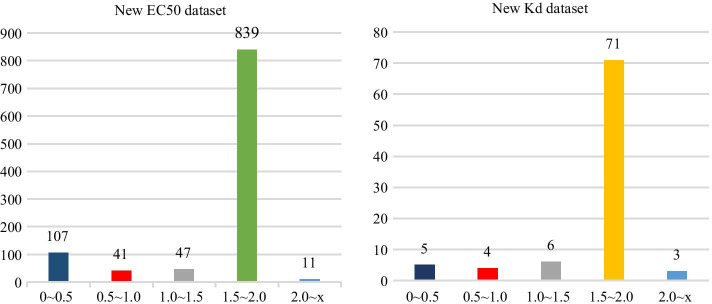
Distribution histogram of |d| for EC50 dataset and Kd dataset

Therefore, we obtained the distribution histogram of |d| (Fig. 6) in new EC50 and Kd datasets, reflecting prediction capability of RF models.

The predictive values of RF models were all greater than zero, suggesting that drug molecule-target interactions do exist, which is consistent with data information gathered from datasets. This indicated that optimal models constructed in this paper could be accurately used for qualitative prediction of DTIs. However, as shown in Fig. 6, eighty percent of |d| distribution was 1.5–2.0. The range of differences was within 2.0 for 98.95% (EC50) 96.63% (Kd) of |d| respectively. This indicated that there is error between predicted value and experimental value. The above demonstrated that quantitative RF prediction model developed in this paper can predict affinity of DTIs to a certain extent based on Kd and EC50.

### Comprehensive comparisons of models

Besides evaluation of application of RF models, comprehensive comparisons were made with predictive models for DTIs previously reported. In recent years, there have been many reports for predicting DTIs, such as Xie L, et al., that integrated transcriptomic data by a deep-learning algorithm to predict the potential DTIs [41]. Olyan R S, et al., developed a novel method based on RF model to improve DTIs prediction accuracy [42]. Chen N, et al., carried out a quantitative analysis of antioxidant activity of antioxidant tripeptides in free radical systems based on QSAR [43]. In above analysis, there are only analysis based on structure of ligand or receptor, rather than taking ligand-receptor as a whole system for DTIs analysis. These methods of analysis, with separated ligands from receptors, can be limited by their own structure and produce non-reciprocal results, leading to poor accuracy. Conversely, in this paper, the models were constructed to take full account of ligands and receptors. From perspective of taking molecule-target as a whole system, we integrated molecule-target descriptors to construct predictive models for DTIs affinity, which is able to avoid unequal results based on receptors or ligands only, thus increasing accuracy of prediction models. At the same time, based on whole system of ligand-receptor, we can collect a large amount of molecule-target data rather than building for a specific target or several targets, expanding scope of application.

There were related reports on quantitative prediction of DTIs affinity. Based on 9948 DTIs quantified by Ki, 1589 molecular descriptors and 1080 protein descriptors, Shar P A, et al., constructed quantitative prediction models for DTIs using RF and SVM model, respectively [44]. However, the Coefficient of Determination-R^2^ of RF and SVM models in training set are 0.88 and 0.86, at the same time that of RF and SVM models in test set are 0.63 and 0.61, which showed that there exists over-fitting. That is to say, predictive models have low accuracy. The main reasons for that would be improper characterization of drug molecules-targets and lack of feature screening. Considering this situation, in this paper, we screened characteristic descriptors of drug molecules from the perspective of molecular vibrations [35, 38]. Moreover, the analysis based on protein sequences rather than 3D structure of protein can ensure a wide range of applicability of models and its accuracy [39]. Therefore, the SVM and RF models in this paper had good results better than above research. In addition, the two datasets in this paper involved 544 and 778 targets respectively, which guaranteed that the models had some broad applicability. Likewise, Hakime Öztürk, et al., constructed DeepDTA to quantify the affinity of ligands-receptors, in which the results were not ideal. In process of building model, more attention was paid to amount of data and neglecting molecular feature representation. The R^2^ of Convolutional Neural Network (CNN) model was less than 0.70, which was lower than optimal RF models in this paper. The MSE of CNN model was high than 0.194, which was high than that of RF models in this paper (0.119) [45]. Abbasi W A, et al., proposed a sequence-based novel protein binding affinity predictor called ISLAND, in which the SVR model for LA kernel was the best model with R = 0.44, MSE = 6.55 [46]. Above comparative results showed that RF models developed based on Kd and EC50 in this paper can perform quantitative prediction of DTIs affinity more accurately with certain applicability and reliability. Moreover, literature already reported has not characterized drug molecules from the perspective of molecular vibrations. Based on the methods and good results of this paper, it was also shown that parametric characterization based on molecular vibrations is crucial for construction of prediction models for DTIs affinity with more accurately.

### Analysis of molecular descriptors and protein descriptors

Molecular descriptors and protein descriptors are essential for construction of quantitative models for DTIs. Judged on the importance of descriptors, we can obtain feature descriptors that have higher importance in the quantification of DTIs affinity based on EC50 and Kd values, which can help us to analyze the importance of different molecular descriptors for quantification of DTIs and provide us with biological insights. Therefore, in the process of feature screening, we filtered the descriptors according to their importance scores to obtain the important descriptors. Importance score of single feature is equal to (oob_accuracy - oob_accuracy_after_perputation), in which the oob_acc_after_perputation is the accuracy of samples on the singletree count after shuffling the dimensional feature with out_of_bag.

For the EC50 datasets, we obtained the top-ranking molecular descriptors and protein descriptors according to importance scores. The top-ranking protein descriptors and molecular descriptors were shown in Tables 5 and 6. In addition, it can be seen in Additional files 1 and 2 for more information on ranking the importance of molecular descriptions and protein descriptors.

**Table 5:** The top-ranking protein descriptors in EC50 datasets

Protein descriptors	Important scores	Protein descriptors	Important score
[G7.1.1.66]	1.00	[G3.3.4.1.8]	0.99
[G4.1.15.1]	1.00	[G3.3.2.1.19]	0.97
[G4.1.23.3]	1.00	[G5.2.2.13]	0.94
[G4.2.8.1]	1.00	[G3.3.2.1.22]	0.90
[G4.2.11.1]	1.00	[G3.3.4.1.27]	0.93
[G7.1.1.47]	0.98	[G7.1.1.43]	0.92

**Table 6:** The top-ranking molecular descriptors in EC50 datasets

Molecular descriptors	Important scores	Concrete meaning
JGI5	0.96	Mean topological charge index of order 5
minaaSe	0.94	Minimum atom-type E-State: aSea
maxaaS	0.91	Maximum atom-type E-State: aSa
minHsSH	0.91	Minimum atom-type H E-State: -SH
maxssssSn	0.88	Maximum atom-type E-State: > Sn <
nHdsCH	0.87	Count of atom-type H E-State: = CH-
maxsNH2	0.86	Maximum atom-type E-State: -NH2
maxssPH	0.86	Maximum atom-type E-State: -PH-
ETA_Beta_s	0.86	A measure of electronegative atom count of molecule
maxddssSe	0.86	Maximum atom-type E-State: = Se =

As shown in Tables 5 and 6, we retained descriptors with importance scores greater than 0.85 in the feature screening process with maximum value of 1. A higher importance score means that the corresponding descriptor is more important for quantification of DTIs.

Based on Tables 2 and 5, it can be found that protein descriptors with high importance are concentrated in Normalized Moreau-Broto autocorrelation (G3), Moran autocorrelation (G4), Transition-Distribution (G7). It is well known that DTIs include a variety of interaction modes, such as electrostatic interaction, hydrophobic interaction, spatial interaction and hydrogen bond. G3 and G4 are the autocorrelation functions combining above physicochemical properties and they can reflect the action strength of DTIs to some extent [47, 48]. G7 represents the amino acid distribution pattern of a specific structural or physicochemical property along a protein or peptide sequence, which directly influence ligand-receptor interactions and it has been used for recognition of protein folds and prediction of ligand-receptor interactions [48]. To sum up, G3, G4 and G7 descriptors are closely related to DTIs, therefore, they have higher importance scores in feature screening.

As for molecular descriptors, according to Tables 1 and 6, it can be found that molecular descriptors with high importance were concentrated in E-state descriptors. E-state descriptors characterize both topological information of each atom and electronic relationships between atoms in the molecule [49]. The three molecular forces, dispersion, dipole moment and hydrogen bonding, which influence the strength of DTIs affinity, are closely related to the electronic relationships characterized by E-state descriptors [49, 50]. Due to their above natures, E-state descriptors have been widely used in the analysis of DTIs [51]. This suggests that E-state descriptors are a good choice for analyzing and predicting DTIs affinity.

For the Kd datasets, the filtering results of descriptors were generally consistent with the EC50 datasets. The top-ranking molecular descriptors were concentrated in E-state descriptors and the top-ranking protein descriptors were concentrated in Normalized Moreau-Broto autocorrelation (G3), Moran autocorrelation (G4), Transition-Distribution (G7). More information can be seen in supplementary data.

In summary, E-state molecular descriptors associated with molecular vibrations and G3, G4 and G7 protein descriptors are of higher importance in the quantification of DTIs. They are important for the analysis and prediction of DTIs affinity.

All in all, in this paper, the ligand and receptor were used as a whole system to analyze and predict DTIs affinity. But the method will not be limited to the known receptor-ligand interaction space and it enables the identification of new action targets of chemical components and the prediction of active affinities. Although there is a margin of error, it can provide clues and guidance to elucidate the action mechanism of drugs. In addition, it is possible to identify unknown potential compounds for the treatment of diseases based on their relevant targets or to reposition existing drugs.

## Conclusion

In this paper, from perspective of overall systematic of ligand-receptor, through screening descriptors based on molecular vibrations and protein sequences, we obtained optimal models based on RF with more accuracy-widely applicability. This method can provide a reference for DTI’s affinity prediction. It also indicated that describing molecular features based on molecular vibrations, taking drug molecule-target as whole system were reliable approaches for construction of prediction model for DTIs affinity and improving its accuracy. In addition, E-state molecular descriptors associated with molecular vibrations and G3, G4 and G7 protein descriptors are important for the analysis and prediction of DTIs affinity.

## Methods and materials

In this paper, we constructed prediction models for DTIs affinity from the perspective of taking molecule-target as a whole system. Firstly, drug molecules and protein sequences as well as ligand-receptor-Kd/EC50 were screened on the basic of existing databases. Secondly, descriptors of drug molecules and protein sequences were calculated separately, and descriptors associated with molecular vibrations were selected from drug molecule descriptors. Thirdly, based on descriptors obtained in step 2, we constructed Kd and EC50 quantified drug molecule-target feature datasets by taking drug molecule and target as a whole system, respectively. Finally, combining above datasets with machine learning algorithms SVM, RF, ANN for construction of prediction models of DTIs affinity.

### Datasets

This paper carried out construction of prediction model of DTIs affinity, which requires a large amount of data support. The drug molecules (ligands) were collected from open source database: PubChem (https://pubchem.ncbi.nlm.nih.gov/), Drugbank (https://go.drugbank.com/) and ChEMBL (https://www.ebi.ac.uk/chembl/) [52–54]. The target protein sequences (receptor) were collected from open source Uniprot database (https://www.uniprot.org/) [55]. In addition, the Kd and EC50 values used to quantify protein–ligand affinity were also obtained from ChEMBL database. All the data as of 10 June 2020.

### Drug molecules and protein sequence descriptors

Descriptors can effectively solve problem of parametric characterization of drug molecules and protein sequences, which facilitate the construction of predictive models for DTIs affinity. In this paper, using PaDEL to calculate the descriptors of drug molecules [56]. Each descriptor has a specific explanation and we screening descriptors of drug molecules from the perspective of molecular vibrations. In addition, protein sequence descriptors such as peptide composition and dipeptide composition were calculated by using PROFEAT web server (https://bio.tools/profeat) [48, 57].

### Feature selection

The Kd, EC50 datasets and molecular descriptors and protein sequence descriptors were integrated separately to obtain the integrated Kd, EC50 datasets. The feature subsets of integrated Kd, EC50 datasets were obtained by using Boruta algorithm (R 3.5.2 version) in feature selection.

Boruta algorithm flow is as follows [58]: first, the features of feature matrix [**X**] are shuffled to obtain shadow feature [**X**^0^], and the shadow features are stitched together with true features to form a new feature matrix [**Y**]. Then, using the new feature matrix as input to output feature importance and calculate the “Z-score” of true and shadow features. Further, taking the largest “Z score” among shadow features as “Z-max”, and marking the real features with “Z-score” greater than “Z-max” as “Important”. At the same time, marking the real features with “Z-score” significantly smaller than “Z-max” as “Rejected”. Finally, repeating five times until we can obtain all features that are marked as “Important”:

$$\begin{aligned} & \left[ {\mathbf{X}} \right] \supseteq \left[ {{\mathbf{X}}_{{1}} ,{\mathbf{X}}_{{2}} ,{\mathbf{X}}_{{3}} ,...... \, ,{\mathbf{X}}_{{{\text{n}} - {2}}} ,{\mathbf{X}}_{{{\text{n}} - {1}}} ,{\mathbf{X}}_{{\text{n}}} } \right] \\ & \left[ {{\mathbf{X}}^{0} } \right] \supseteq \left[ {{\mathbf{X}}^{{1}} ,{\mathbf{X}}^{{2}} ,{\mathbf{X}}^{{3}} ,...... \, ,{\mathbf{X}}^{{{\text{n}} - {2}}} ,{\mathbf{X}}^{{{\text{n}} - {1}}} ,{\mathbf{X}}^{{\text{n}}} } \right] \\ & \left[ {\mathbf{X}} \right] \to \left[ {{\mathbf{X}}^{{\mathbf{0}}} } \right] \\ & \left[ {\mathbf{Y}} \right] = \left[ {\mathbf{X}} \right] \cup \left[ {{\mathbf{X}}^{{\mathbf{0}}} } \right] \\ & {\text{Feature}}\;{\text{importance}}\left[ {\mathbf{Y}} \right] = {\text{Z - score}} \\ & {\text{If}}\;{\mathbf{X}}^{i} \in \left[ {{\mathbf{X}}^{{\mathbf{0}}} } \right],{\text{Max}}\;{\text{feature}}\;{\text{importance}}\left[ {{\mathbf{X}}^{i} } \right] = {\text{Z - max}} \\ \end{aligned}$$

**X**_***i***_ $\in$[**X**], so **X**_***i***_ $\in$[**Y**], feature importance [**X**_***i***_]** = **Z-score; when Z-score > Z-max, **X**_***i***_ = “Important”; If Z-score < Z-max, **X**_***i***_ = “Rejected”.

**X**_1_ ~ **X**_n_ and **X**^1^ ~ **X**^n^ are the attribute indicators in the feature matrix.

In this paper, Z-score (important score) was defined as 0.6. In addition, some data was marked as “Tentative”, which means importance of the data is not clear. To ensure reliability of feature filtering, we excluded the data marked “Tentative” and “Rejected”.

### The quantitative prediction model for DTIs affinity

The feature subsets was first pre-processed, and then combined with machine learning algorithms for construction of quantitative prediction models for DTIs affinity.

#### Pre-processing of feature subsets of descriptors

We normalized descriptors of the feature subsets in the range from -1 to 1. Meanwhile, the EC50 and Kd values that quantify DTIs affinity were processed in logarithmic form-Log_2_ (Kd), Log_2_ (EC50). In other words, we obtained feature subsets in which took Log_2_ (Kd) and Log_2_ (EC50) values characterize drug molecule-target affinity, respectively.

#### Construction of quantitative prediction model for DTIs affinity

The subsets obtained by feature selection were combined with random forest (RF) [59], support vector machine (SVM) [60] and artificial neural network (ANN) [61] to construct quantitative prediction model of DTIs affinity respectively. On the basic of ten-fold cross-validation, the feature subsets were randomly and equally divided into 10 data sets, where 9 groups of data were rotated as training sets for model construction, and the remaining 1 group of data will be used as a test set for model validation.

### Evaluation and application of quantitative prediction model for DTIs affinity

The train and test validation were made use to wholly assess these models. Briefly, (1) the feature subsets were divided into 10 subsets randomly and equally as mentioned previously and 9 subsets were selected as training sets for modeling while the remaining subset served as test set for validating models. This process was repeated ten times until every subset served as test set. (2) Using different test sets to exert ten external independent validation. The nature of quantitative prediction models for DTIs affinity is regression models. Therefore, we used the Error Sum of Squares (SSE), Mean Square Error (MSE), Mean Absolute Error (MAE), Relative Mean Square Error (RMSE) and Coefficient of Determination (R^2^) to evaluate the performance of models. The evaluation parameters can be expressed in the form as follows:

$$SSE = \sum \left( {Y\_actual - Y\_predict} \right)^{2}$$

$$MSE = \frac{1}{n}\mathop \sum \limits_{i - 1}^{n} \left( {Y\_actual - Y\_predict} \right)^{2}$$

$$RMSE = \sqrt {\frac{1}{n}\mathop \sum \limits_{i - 1}^{n} \left( {Y\_actual - Y\_predict} \right)^{2} }$$

$$MAE = \frac{1}{n}\mathop \sum \limits_{i = 1}^{n} \left| {Y\_actual - Y\_predict} \right|$$

$$R^{2} = 1 - \frac{{\sum \left( {Y\_actual - Y\_predict} \right)^{2} }}{{\sum \left( {Y\_actual - Y\_mean} \right)^{2} }}$$

*Y*_-_*actutal* and *Y*_-_*predict* denoted experimental value and predicted value, respectively. *n* is number of samples in the training sets or test sets. A higher R^2^ value means model is more reliable. A lower MSE or SSE value means that model has higher accuracy.

Through above parametric evaluation to select the optimal models to predict quantitative affinity between drug molecules and targets collected in Binding DB database and comprehensive comparison were made with predictive models for DTIs affinity previously reported.

## Supplementary Information

**Additional file 1**. Molecular descriptor information.

**Additional file 2**. Protein descriptor information.

**Additional file 3**. Implications of molecular descriptions related to molecular vibrations.

## Data Availability

The algorithm processing and applications involved in this paper were all done in R (version 3.5.2). All data and materials such as raw data, EC50 dataset, Kd dataset, feature datasets, software, code, etc. used for quantitative prediction model construction and research results in this paper are available on open source data repository-Zenodo.org (https://zenodo.org/) with Zenodo_ numbers: 4699610 and 5510335.

## Abbreviations

ANN: Artificial neural network
DTIs: Drug–target interactions
MAE: Mean absolute error
MD: Molecular docking
MSE: Mean square error
QSAR: Quantitative structure–activity relationship
R^2^: Coefficient of determination
RF: Random forest
RMSE: Relative mean square error
SSE: Error sum of squares
SVM: Support vector machine
